# Totally laparoscopic versus laparoscopic-assisted total gastrectomy for upper and middle gastric cancer: a single-unit experience of 253 cases with meta-analysis

**DOI:** 10.1186/s12957-016-0860-2

**Published:** 2016-03-31

**Authors:** Ke Chen, Yu Pan, Jia-Qin Cai, Di Wu, Jia-Fei Yan, Ding-Wei Chen, Hong-Mei Yu, Xian-Fa Wang

**Affiliations:** Department of General Surgery, Sir Run Run Shaw Hospital, School of Medicine, Zhejiang University, 3 East Qingchun Road, Hangzhou, 310016 Zhejiang Province China

**Keywords:** Gastric cancer, Total gastrectomy, Intracorporeal anastomosis, Laparoscopy, Meta-analysis

## Abstract

**Background:**

Laparoscopic-assisted total gastrectomy (LATG) is the most commonly used methods of laparoscopic gastrectomy for upper and middle gastric cancer. However, totally laparoscopic total gastrectomy (TLTG) is unpopular because reconstruction is difficult, especially for the intracorporeal esophagojejunostomy. We adopted TLTG with various types of intracorporeal esophagojejunostomy. In this study, we compared LATG and TLTG to evaluate their outcomes.

**Methods:**

From March 2006 to September 2015, 253 patients with upper and middle gastric cancer underwent laparoscopic total gastrectomy (LTG), 145 patients underwent LATG, and 108 patients underwent TLTG. The clinicopathological characteristics and postoperative outcomes were retrospectively compared between the two groups. Furthermore, a systematic review and meta-analysis were conducted.

**Results:**

The operation time and estimated blood loss were similar between the groups. There were no significant differences in first flatus, diet initiation, and postoperative hospital stay. The surgical complication rates were 17.2 % (25/145) and 13.9 % (15/108) in the LATG and TLTG groups, respectively. The meta-analysis also revealed no significant differences in the operation time, estimated blood loss, time to first flatus, length of hospital stay, overall, and anastomosis-related complications among the groups.

**Conclusions:**

TLTG is a feasible choice for gastric cancer patients, with comparable results to the LATG approach.

## Background

Gastric cancer is the fourth most common cancer worldwide and the second most frequent cancer-related cause of death in 2008 [1]. Surgery has been widely performed as the most effective treatment for resectable gastric cancer. Ever since it was first reported in 1994, the number of patients undergoing laparoscopic gastrectomy (LG) has been rapidly increasing. A randomized controlled trial has showed that laparoscopic gastrectomy is not inferior to open gastrectomy in patients with early distal gastric cancer. Large retrospective studies have also obtained acceptable oncologic outcomes [2]. In addition, laparoscopic surgery has the advantages of faster recovery, fewer complications, reduced hemorrhaging that reduces the likelihood of needing a blood transfusion, a smaller incision that reduces pain, the probability of intestinal obstruction, and the risk of wounding. Laparoscopic-assisted gastrectomy (LAG) and totally laparoscopic gastrectomy (TLG) are two common methods of LG for gastric cancer. Usually, extracorporeal anastomosis with LAG was performed through a 5–7-cm small incision in the middle upper abdomen. However, extension of the laparotomy is often necessary to obtain a better view for secure anastomosis in obese patients. Furthermore, the procedure is more difficult in cases with a short esophageal stump, because of the limited workspace even worse than larger laparotomy. TLG was established as a method for the intracorporeal resection and anastomosis using the laparoscopic technique. It has advantages over LAG, including a smaller wound and is less invasive [3–6].

Although the amount of totally laparoscopic distal gastrectomy (TLDG) performed for gastric cancer has gradually increased due to advancements in laparoscopic surgical instruments and the accumulation of operative experience, total laparoscopic total gastrectomy (TLTG) is not widely performed because of its technical difficulty, especially for the intracorporeal esophagojejunostomy.

Based on lots of laparoscopic experience from different laparoscopic operations, such as pancreatic and gastric surgery [7–13], we were encouraged to develop TLTG with different styles of interacorporeal esophagojejunostomy to treat middle and upper gastric cancer. This article compares the short-term achievements of patients who experience TLTG and laparoscopic-assisted total gastrectomy (LATG) in our center. A systematic review and meta-analysis were also conducted to further clarify the feasibility and safety of TLTG and to summarize the operative experience.

## Methods

### Patients

Between March 2006 and September 2015, 253 patients with middle or upper gastric carcinoma underwent laparoscopic total gastrectomy (LTG) at the Department of Gastrointestinal Surgery at the Sir Run Run Shaw Hospital, Affiliated Hospital of School of Medicine, Zhejiang University, China. The patients were divided into two groups according to reconstructive method, such as intracorporeal or extracorporeal reconstruction. All 253 patients were preoperatively examined by esophagogastroduodenoscopy (with biopsy), abdominal and pelvic computed tomography (CT), chest radiography, electrocardiography, and basic blood testing. Endoscopic ultrasonography (EUS), liver magnetic resonance imaging (MRI), or chest CT was selectively performed as appropriate.

The patients’ surgical characteristics (operative time, intraoperative hemorrhage), postoperative recovery (time to first flatus, time to initiate oral intake, complications, and length of postoperative hospital stay), and histopathologic indices (number of resected lymph nodes, surgical margins distance) were observed and compared between the two groups. Postoperative complications were classified as medical (cardiovascular, respiratory, or metabolic events; nonsurgical infections; deep venous thrombosis; and pulmonary embolism) or surgical (any anastomotic leakage or fistula, any complication that required reoperation, intra-abdominal collections, wound complications, bleeding events, pancreatitis, ileus, delayed gastric emptying, and anastomotic stricture). The Institutional Review Board of Sir Run Run Shaw Hospital of Zhejiang University approved this study protocol, and written informed consent was obtained from all patients before the investigation.

### Surgical procedure

The patients underwent LTG with modified D_2_ lymph node dissection and Roux-en-Y reconstruction for gastric cancer. We previously performed LATG by using an anvil inserted via minilaparotomy. A policy of TLG was adopted at our hospital from its inception because we considered that it would confer several advantages. Therefore, we started performing TLTG using an anvil and intracorporeal purse-string suture technique in November 2007 and started using intracorporeal hand-sewn esophagojejunostomy in September 2012. Generally speaking, there are two approaches of intracorporeal esophagojejunostomy, including mechanical staplers and hand-sewn purse-string suture techniques. The details of the surgery are described in our previously published articles [14].

### Systematic review and meta-analysis

We searched three major electronic databases (PubMed, EMBASE, and the Cochrane Library) for literature comparing LATG and TLTG published between January 1995 and September 2015. The following keywords were used: “laparoscopy,” “laparoscopic,” “gastric cancer,” and “gastrectomy.” The language of the articles was limited to English. Review articles, opinion pieces, and articles with no control group were excluded. Two investigators reviewed the titles and abstracts and assessed the full text to establish eligibility, and disagreements were resolved via discussion. The Newcastle-Ottawa Quality Assessment Scale was used for quality assessment of the observational studies. A threshold of six stars or above has been considered indicative of high quality.

### Statistical analysis

All statistical analyses were performed using the Statistical Package for the Social Sciences (SPSS®) version 16.0 (SPSS, Inc. Chicago, IL, USA). The differences in the measurement data were compared using the Student’s *t* test, and comparisons between groups were tested using a the χ^2^ test or the Fisher exact probability test. For the meta-analysis, Review Manage Version 5.1 (RevMan 5.1) software downloaded from Cochrane Library was used. Continuous variables were assessed using weighted mean difference (WMD), and dichotomous variables were analyzed using the risk ratio (RR). The anastomosis-related complications included anastomotic leakage, hemorrhage, and stricture or stenosis. To account for clinical heterogeneity, which refers to diversity in a sense that is relevant for clinical situations, we used the random-effects model based on DerSimonian and Laird methods. Potential publication bias was determined by conducting informal visual inspections of the funnel plots based on the complications. *P* < 0.05 was considered to be statistically significant.

## Results

### Baseline characteristics

Table 1 summarizes the baseline characteristics of the two study groups. Of all 253 patients, 145 underwent LATG, while TLTG was performed on the other 108 patients. Both groups were well balanced for the variables (age, gender, BMI, comorbidity, ASA score, tumor size, tumor location, and TNM stage).

**Table 1:** Comparison of the clinicopathological characteristics

		LATG (*n* = 145)	TLTG (*n* = 108)	*P* value
Age (years)		57.3 ± 12.5	59.4 ± 11.1	0.18
Gender	Male	98	73	0.99
	Female	47	35	
BMI index (kg/m^2^)		23.1 ± 4.2	23.5 ± 3.5	0.42
Comorbidity	Absence	97	76	0.56
	Presence	48	32	
ASA classification	I	80	65	0.60
	II	58	37	
	III	7	6	
Tumor size (cm)		4.3 ± 2.0	4.0 ± 1.8	0.23
Tumor location	Middle	36	33	0.31
	Upper	109	75	
Histology	Differentiated	84	67	0.51
	Undifferentiated	61	41	
TNM stage	IA/IB	54/28	28/25	0.45
	IIA/IIB	18/9	14/13	
	IIIA/IIIB/IIIC	12/10/14	13/7/8	

### Surgical outcomes in the LATG and TLTG groups

Table 2 summarizes the operative outcomes and hospital courses of the LATG and TLTG groups. The operation time (234.8 ± 48.5 min versus 225.6 ± 52.7 min, *P* = 0.15) was similar between the groups. However, the anastomotic times were lower in the LATG group than those in the TLTG group (32.8 ± 19.5 min versus. 47.5 ± 23.2 min, *P* < 0.01). The mean blood loss was lower in the TLTG group than those in the LATG group but these differences were not significant (137.6 ± 54.7 mL versus. 125.3 ± 62.8 mL, *P* = 0.10). The proximal margin distance and number of retrieved lymph nodes were not significantly different between the two groups. Postoperative outcomes include the first flatus time (3.4 ± 1.0 days in the LATG versus 3.4 ± 1.1 days in the TLTG, *P* = 0.19), diet start time (4.5 ± 1.3 days versus 4.4 ± 1.4 days, *P* = 0.56), and duration of postoperative hospital stay (9.4 ± 2.5 days versus 9.2 ± 3.0 days, *P* = 0.56).

**Table 2:** Comparison of surgical outcomes and postoperative recovery

	LATG (*n* = 145)	TLTG (*n* = 108)	*P* value
Operation time (min)	234.8 ± 48.5	225.6 ± 52.7	0.15
Anastomotic time (min)	32.8 ± 19.5	47.5 ± 23.2	<0.01
Estimated blood loss (mL)	137.6 ± 54.7	125.3 ± 62.8	0.10
Harvested lymph nodes	31.2 ± 10.4	32.8 ± 8.9	0.20
Proximal resection margin (cm)	4.3 ± 1.7	4.6 ± 1.6	0.16
First flatus (days)	3.4 ± 1.0	3.4 ± 1.1	0.19
Diet start time (days)	4.5 ± 1.3	4.4 ± 1.4	0.56
Postoperative hospital stay (days)	9.4 ± 2.5	9.2 ± 3.0	0.56

Table 3 shows the postoperative complications in the two groups. The postoperative complications are listed in Table 3. There was no in-hospital mortality and 30-day mortality. Complications developed in 17.2 % (25/145) of patients in the LATG group and 13.9 % (15/108) of patients in the TLTG group. There were no significant differences in overall postoperative complications, surgical complications, or medical complications between the two groups.

**Table 3:** Comparison of postoperative complications

	LATG (*n* = 145)	TLTG (*n* = 108)	*P* value
Total complication	25	15	0.42
Surgical complications	20	13	0.63
Anastomotic leakage	1	1	
Anastomotic stricture	2	3	
Intracorporeal hemorrhage	1	2	
Abdominal abscess	4	1	
Stasis	3	2	
Pancreatic leakage	2	1	
Ileus	3	1	
Lymphorrhea	1	1	
Wound infection	3	1	
Medical complications	5	2	0.43
Pulmonary embolism	0	1	
Pulmonary infection	4	1	
Deep venous thrombosis	1	0	

### Outcomes of a systematic review and meta-analysis

A total of 86 articles were identified in the three major electronic databases using the aforementioned search strategy. The titles and abstracts were reviewed, and articles without comparison of LATG and TLTG were excluded. Only three articles remained [15–17]. Including the present data, there were 816 participants in three studies (237 patients in the LATG group and 355 patients in the TLTG group). According to the Newcastle-Ottawa Quality Assessment Scale, all three studies received eight stars. The characteristics and methodological quality assessment scores of the included studies are shown in Table 4.

**Table 4:** Characteristics of included studies

Author	Nation	Study type	Publication year	Study period	Sample size		Quality scores	Details of IE
					LATG	TLTG		
Kim	Korea	Pros	2013	2010–2011	23	90	6	Functional end-to-end
Jung	Korea	Retro	2013	2004–2012	47	40	6	OrVil™
Ito	Japan	Pros	2014	2001–2012	46	117	6	OrVil™

*Retro* retrospective observational study, *Pros* prospective observational study, *IE* intracorporeal esophagojejunostomy

**Fig. 1:**
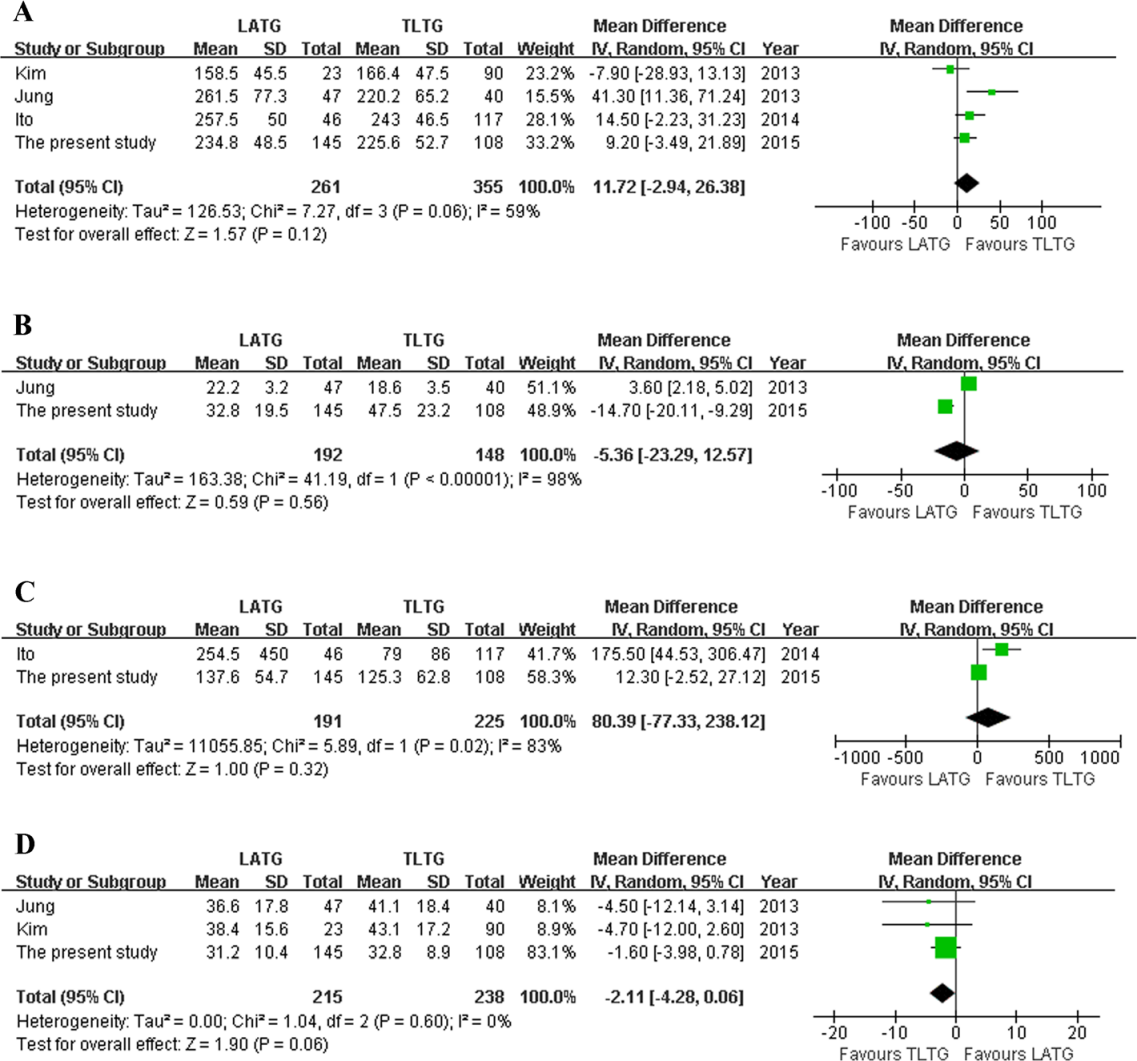
Meta-analysis of the pooled data. **a** Operation time. **b** Anastomotic time. **c** Blood loss. **d** Harvested lymph nodes

The results of meta-analysis are summarized in Table 5. Meta-analysis of the operation time (WMD = 11.72 min, 95 % confidence interval (CI) −2–94 to 26.38, *P* = 0.12) (Fig. 1a) and anastomotic time showed no significant difference between the two groups (WMD = −5.36 min, 95 % CI −23.29 to 12.57, *P* = 0.56) (Fig. 1b). There was also no significant intraoperative blood loss difference between the two groups (WMD = 80.39 ml, 95 % CI −77.33 to 238.12, *P* = 0.32) (Fig. 1c).

**Table 5:** Pooled short-term outcomes of meta-analysis

Outcomes	Number of studies	Sample size		Heterogeneity (*P, I* ^*2*^)	Overall effect size	95 % CI of overall effect	*P* value
		LATG	TLTG				
Operation time (min)	4	261	355	0.06, 59 %	WMD = 11.72	−2.94~26.38	0.12
Anastomotic time (min)	2	192	148	<0.01, 98 %	WMD = −5.36	−23.29~12.57	0.56
Blood loss (ml)	2	191	225	0.02, 83 %	WMD = 80.39	−77.33~238.12	0.32
Harvested lymph nodes	3	215	238	0.60, 0 %	WMD = −2.11	−4.28~0.06	0.06
Proximal margin (cm)	3	215	238	0.26, 26 %	WMD = −0.06	−0.37~0.26	0.73
First flatus (days)	3	215	238	0.44, 0 %	WMD = −0.01	−0.19~0.16	0.88
Diet start time (days)	3	215	238	0.12, 53 %	WMD = 0.37	−0.15~0.90	0.17
Hospital stay (days)	3	215	238	0.63, 0 %	WMD = 0.32	−0.31~0.96	0.32
Overall complications	2	168	198	0.71, 0 %	RR = 1.31	0.78~2.20	0.30
Anastomosis-related complications	4	261	355	0.46, 0 %	RR = 1.26	0.60~2.65	0.55

*WMD* weighted mean difference, *RR* risk ratio

The length of proximal resection margin was similar for both groups. However, the number of harvested lymph nodes of TLTG was more than that of LATG with a marginal difference (WMD = −2.11 cm; 95 % CI −4.28 to 0.06, *P* = 0.06) (Fig. 1d).

**Fig. 2:**
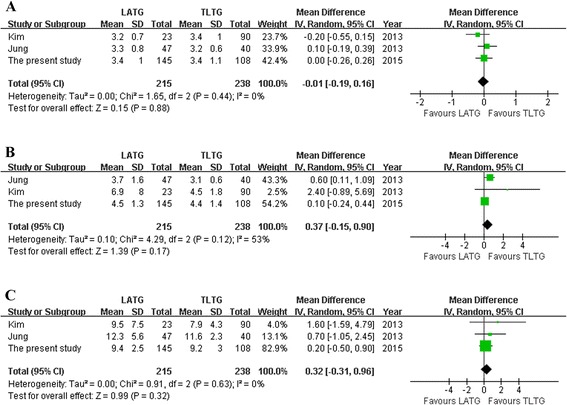
**a** First flatus. **b** Diet start time. **c** Hospital stay

**Fig. 3:**
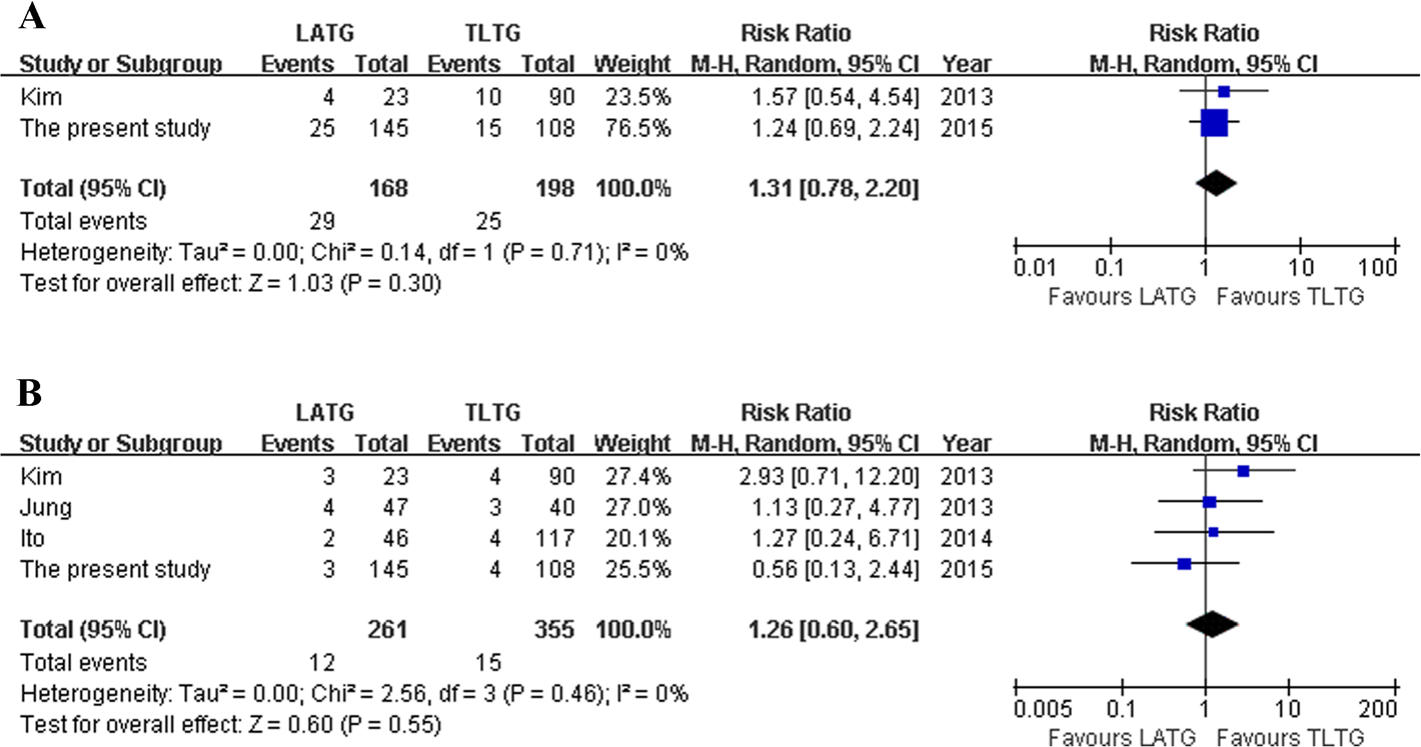
**a** Overall complications. **b** Anastomosis-related complications

**Fig. 4:**
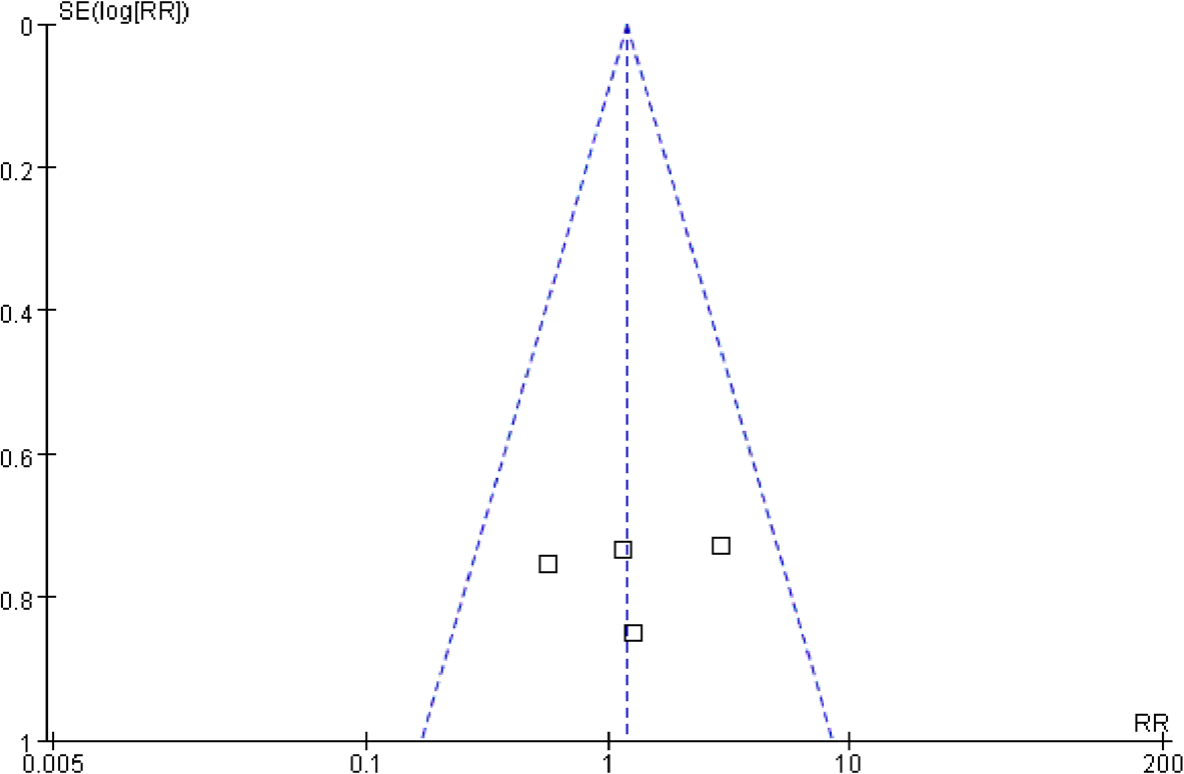
Funnel plot of the anastomosis-related complications

With regard to postoperative recovery outcomes, such as time to flatus and oral intake and duration of postoperative hospital stay, all outcomes showed no significant difference between the two groups (Fig. 2). Besides, none of the included studies reported mortality, and the overall and anastomosis-related complications were similar between the groups (Fig. 3). Visual inspection of the funnel plot of the anastomosis-related complications revealed symmetry, indicating no serious publication bias (Fig. 4).

## Discussion

Although laparoscopic surgery is frequently performed for the treatment of gastric cancer and LATG has been a very common approach, TLTG is not widely performed because of its technical difficulty. Esophagojejunal anastomosis after TLTG is one of the difficult procedures because of the difficulties in purse-string suturing and anvil placement. Moreover, concerns about the potential for high morbidity and mortality rates limited the enthusiasm for intracorporeal esophagojejunostomy. With recent advancements of laparoscopic instruments and the accumulation of operative experience, esophagojejunostomy can now be completed laparoscopically. In this study, we compared the results of TLTG to LATG in contemporary patient cohorts at a single institution over the same time period with the diagnosis of a gastric cancer. We included our initial experience with TLTG and found that outcomes were still quite comparable to the LATG approach.

The safety of operation is an important focus for surgeons who perform TLTG. In our research, only 15 patients (13.9 %) suffered from postoperative complications. Both the results of meta-analysis and our data showed that the similar complications occurred in the two groups. Based on the further analysis of anastomosis-related complications, such as stenosis, anastomotic leakage, and hemorrhage, among 108 patients, only 4 patients suffered from complications that were directly connected with anastomosis in the group of TLTG. Therefore, the rate of the complications related to anastomosis was 3.7 % with no significant difference by data of our center or meta-analysis.

Anastomotic leakage was one of the most frequent reconstruction-related complications and occurred in 1.1 %, which is comparable to the ratio in open surgery. The leaks, followed by intra-abdominal abscess, required no intervention. Linear stapler side-to-side esophagojejunostomy could reduce anastomotic stenosis, because a stoma larger than 30-mm diameter can be created, when 45-mm staplers are used [18]. This is one of the advantages of the linear-stapled method over circular stapling with regard to reducing anastomotic stricture. The reduced risk of anastomotic stenosis could contribute to a better quality of life for patients, because the symptoms of stenosis are one of the most important factors impairing the quality of life in patients after gastrectomy. However, the esophagojejunostomy made by linear or circular stapler could increase the risk of anastomotic hemorrhage. In our center, we started using intracorporeal hand-sewn esophagojejunostomy in September 2012, and there were no anastomotic hemorrhages after that.

Because the reconstruction portion of TLTG can be difficult, some researchers believe that longer operation time adversely affects patient outcome. In our current study, the operation time for TLTG was not longer than LATG. On the basis of our TLTG experience, two points contribute to this: First, the procedure of anastomosis was simplified using modified intracorporeal esophagojejunostomy techniques. Second, the opening and closure of minilaparotomy are exempted in the TLTG, which can shorten the operation time by 15 min. Certainly, the learning curve also effects on the operation time. What is more, skillful surgeons are capable of performing the operation safer and faster than unskilled surgeons.

Compared with the incision at the epigastrium required by LATG, the incision in TLTG is smaller. Therefore, TLTG is the better cosmetic solution. However, it is not clear whether TLTG is really less invasive than LATG or provides many clinical benefits to the patient in addition to cosmetic factors. Based on our data, the intraoperative blood loss in the group of TLTG was less than that in the group of LATG, but this value was not statistically significant. In the group of LATG, blood loss might increase due to skin incision and anastomosis through small skin incision by hand manipulation. Moreover, the esophageal stump must be pulled out from the abdominal cavity when LATG is conducted. The pulling puts great pressure on the esophageal stump and might even cause tearing and bleeding of the spleen envelope. However, this result should be interpreted prudently for the variation in blood loss between studies was high, with heterogeneity as a result of different methods of estimating blood loss.

Oncological results critically measure the success of laparoscopic surgery of malignant tumors. With short follow-up times, the main indicators of oncological quality are numbers of retrieved lymph nodes and surgical resection margin. It is our opinion that a technically similar oncologic resection can be performed regardless of whether the LATG or TLTG approach is used. As such, we would argue that neither procedure is technically superior nor that harvesting an adequate number of lymph nodes is largely dependent on the technique of the surgeon and on pathologic analytic variability. However, the meta-analysis demonstrated that the number of harvested lymph nodes of TLTG was more than that of LATG with a marginal difference (*P* = 0.06). However, in the included studies and our center, surgeons perform LATG during their early period and TLTG during the late period. The amount of dissected laparoscopic lymph nodes closely connects with the surgical skills. And thus, such time difference seemed to connect to the clinically obvious differences in the resection of lymph nodes.

In recent years, various modified intracorporeal esophagojejunostomy techniques have been reported, such as laparoscopic purse-string suture technique using Endo Stitch (Covidien) [19], Endo-PSI (Hope Electronics) [20], or a hemi-double stapling technique [21]. Another two intracorporeal reconstruction methods may be most representative; one using a transorally inserted anvil (OrVil; Covidien) to make an end-to-side esophagojejunostomy [22], the other using linear staplers to make a side-to-side anastomosis [23]. However, the optimal method for esophagojejunostomy in LTG remains to be established. The OrVil method carries the possible risk of pharyngeal or esophageal injury resulting from the passage of the anvil head at the level of the tracheal bifurcation. This can result in abdominal infection because of a contaminated OrVil tube and an overlapping anastomosis line on the esophagojejunostomy site. It is reported that TLTG with linear-stapled anastomosis is a simple, feasible choice with less postoperative reconstruction-related complications [18]. It has the additional limitation of requiring a sufficient esophageal length and necessitates taking down more of the esophagus within the mediastinum. In particular, when the more proximal esophagus needs to be transected, manipulation using an endoscopic linear stapler becomes extremely difficult. The traditional approach of circular stapler anvil is also limited for the placement of circular staplers which are improper in the laparoscopic surgery because of the absence of a matching tube and bigger size. The pneumoperitoneum was vulnerable to the placement, so the vision is unclear. The limitations resulted from the mechanical approach are overcome by hand-sewn esophagojejunostomy. The process of suture should be clearly noticed under high-definition laparoscopy, which makes anastomosis reliable. Besides, the operating space is large, and there is no tension in the whole anastomosis procedure. Also, this method does not need a longer esophageal stump. However, hand-sewn method requires the operators with rich experience in laparoscopic suture procedures, and it may take more time. Based on our experience, the linear stapler should be adjusted for patients with the lesions in the lower cardia and body as well as the fundus of the stomach. As for the patients with the lesions in the middle and upper cardia, the circular stapler can be selected to accommodate the surgical margin. Finally, if the surgeon is experienced in laparoscopic hand-sewn technique, and it can be applied after total gastrectomy, no matter where the tumor is located.

There are several limitations to our studies. First, all of the outcomes resulted from East Asia, where the average BMI is lower than a common Western BMI. However, our results would be also suitable for Western patients, because intracorporeal reconstruction is easier than reconstruction through minilaparotomy in obese patients. Second, there is a difference in the time period when each of the surgical procedures was performed. LATG has been performed since March 2006, whereas TLTG has been performed since November 2007. Various operative factors related to the procedure itself, such as surgical instruments, sutures, and drugs, may have influenced the results. In addition, there may be differences in operator skill and perioperative care protocols among the surgical groups. Third, the majority of the studies analyzed focused only on total gastrectomy. However, the included studies had cases of proximal gastrectomy because the sample size of the remaining studies is too small for definitive conclusions and the larger the number of patients in a meta-analysis, the greater its power to detect a possible treatment effect [16]. Therefore, we did not exclude the study. Although, such a low number does not imply a significant bias, it still can lead to clinical heterogeneity.

## Conclusions

The current study showed that TLTG is a feasible choice for gastric cancer patients, with comparable results to the LATG approach. However, more methodologically high-quality comparative studies are required to adequately evaluate the status of TLTG.

## Abbreviations

CT: computed tomography
EUS: endoscopic ultrasonography
LAG: laparoscopic-assisted gastrectomy
LATG: laparoscopic-assisted total gastrectomy
LTG: laparoscopic total gastrectomy
MRI: magnetic resonance imaging
RR: risk ratio
TLDG: totally laparoscopic distal gastrectomy
TLG: totally laparoscopic gastrectomy
TLTG: totally laparoscopic total gastrectomy
WMD: weighted mean difference
